# Enabling technologies for *in situ* biomanufacturing using probiotic yeast

**DOI:** 10.1016/j.addr.2025.115605

**Published:** 2025-05-16

**Authors:** William Parker, Amanda Taylor, Aryan Razdan, Jose Escarce, Nathan Crook

**Affiliations:** Department of Chemical Engineering, North Carolina State University, Raleigh, NC 27695, USA

**Keywords:** Yeast, Synthetic Biology, Probiotics, Therapeutics, Drug Discovery

## Abstract

*Saccharomyces boulardii* (*Sb*) is a Generally Regarded As Safe (GRAS) probiotic yeast currently used to alleviate symptoms from various gastrointestinal diseases. *Sb* is a promising platform for probiotic and biotherapeutic engineering as it is the only probiotic eukaryote and carries with it a unique set of advantages compared to bacterial strains, including resistance to phage, high protein secretion abilities, and intrinsic resistance to antibiotics. While engineered *Sb* has not been studied as extensively as its close relative *Saccharomyces cerevisiae* (*Sc*) many genetic engineering tools developed for *Sc* have also shown promise in *Sb*. In this review, we address recent research to develop tools for genetic engineering, colonization modulation, biomarker sensing, and drug production in *Sb*. Ongoing efforts, especially those that overcome gut-specific challenges to engineered performance, are highlighted as they advance this chassis as a scalable platform for treating gastrointestinal diseases.

## GRAPHICAL ABSTRACT

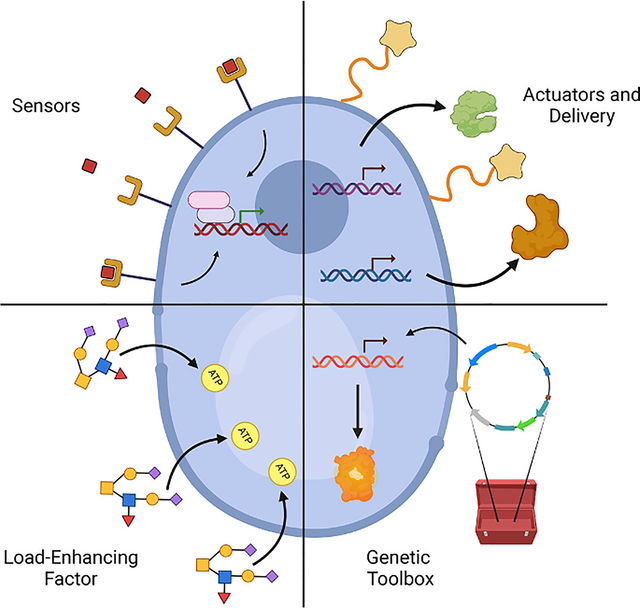

## 1. Introduction

Probiotics are microorganisms that, when administered in adequate amounts, benefit the host’s health [1]. *Saccharomyces boulardii* (*Sb*) is a Generally Regarded As Safe (GRAS) probiotic yeast discovered by Henri Boulard in the early 20th century during travels in southeast Asia [2]. Studies demonstrate *Sb* alleviates diarrhea [3–6] or diarrhea-like symptoms in Crohn’s disease [7], irritable bowel syndrome (IBS) [8], and ulcerative colitis [9]. These activities, combined with its ease of manufacture, have made this yeast popular in supplements and probiotic-containing foods.

*Sb*’s closest relative is the well-studied *Saccharomyces cerevisiae* (*Sc*). In fact, genomes of these strains are so similar that *Sb* is more correctly described as a substrain of *Sc* rather than a separate species. However, unlike most laboratory *Sc* strains*, Sb* can tolerate low pH and grows optimally at 37 °C, the human body temperature [10,11]. Other crucial distinctions are that *Sb* cannot sporulate or consume galactose. Researchers have begun to unravel the genotypic bases for these traits [10]. For example, seven SNP and eight indel mutations have been identified in the gene encoding *Sb*’s a-factor (*MATa*), which collectively are hypothesized to be responsible for *Sb’*s non-sporulating phenotype [12]. In turn, the inability to sporulate is thought to be responsible for the complete lack of several retrotransposon families in the *Sb* genome [12]. It has also been recently shown that premature stop codons in *Sb*’s *PGM2* gene are responsible for its lack of galactose metabolism [13]. Furthermore, *Sb* has greater copy numbers of genes involved in protein synthesis and the stress response [12], which may enable *Sb* to adapt to stressful conditions under nitrogen deprivation via mechanisms such as pseudo-hyphal switching [14]. *Sb* also expresses several proteins responsible for forming flocs, which may be truncated in low-flocculating strains of *Sc* [12]. *Sb*’s ability to form flocs is thought to contribute to its antimicrobial properties by enabling it to adhere to pathogens before they can adhere to the cells in the intestine. Additionally, *Sb* encodes proteases and phosphatases that cleave *Clostridiodes difficile* toxins, making it a particularly well-suited candidate to treat *C. difficile* infections [10,15,16]. *Sb* also produces acetic acid under aerobic conditions at human body temperature and has a thicker cell wall, which may explain its acid resistance and antimicrobial properties in regions of the GI tract with higher oxygen levels [10,17]. Taken together, the high relatedness of *Sb* and *Sc* has allowed dissection of *Sb’s* unique phenotypes.

In addition to its native properties, *Sb* is an attractive platform for *in situ* and *ex situ* biomanufacturing via genetic engineering, and has several unique aspects that distinguish it from probiotic bacteria, including lactic acid bacteria and *E. coli* Nissle. For example, *Sb* can perform post-translational modifications (PTMs), readily secrete recombinant proteins at high titers, unlike most bacteria. Its inability to sporulate or mate makes *Sb* unlikely to transfer genes to other microbes, which is a prominent concern for engineered bacteria. Also, unlike bacteria, *Sb* is intrinsically resistant to bacteriophage and unaffected by antibiotics, which allows for administration to patients receiving antibiotics or phage therapies [2]. Like bacteria, *Sb* can also colonize gnotobiotic mice [18], and protocols for enhancing colonization in conventional mice are well-established [19,20], making it ideal for preclinical studies. From a patient compliance perspective, *Sb* retains its viability when freeze-dried within capsules and unlike some bacteria (e.g. *E. coli* Nissle) is palatable as a liquid suspension. These characteristics provide a sound basis for utilizing *Sb* as a chassis in future biotherapeutic applications. *Sb*’s resistance to temperature and acid also makes it a strong platform for fermenting pharmaceuticals, from small-molecule drugs to biologics. Despite these advantages, no engineered *Sb* strains (or other microbes for that matter) have yet made their way to the clinic, making a realistic appraisal of current engineering efforts particularly timely.

In this review, we will summarize and comment on recent developments in engineering this emerging *Sb* probiotic chassis for drug delivery to the human intestine. We follow a bottom-up approach, describing *Sb*’s growing genetic toolbox and subsequently describing approaches for improving *Sb*’s ability to colonize the gut, sense its surroundings, and ultimately treat disease. Important foundational and recent studies are presented, highlighting key barriers that must be overcome to accelerate *Sb*’s therapeutic potential and proposing several new approaches toward this goal.

## 2. The genetic toolbox for *Sb*

Reliable genetic tools are a prerequisite for engineering *Sb* to deliver biotherapeutics. Fortunately, many genetic tools developed for *Sc* have been adapted for work in *Sb*, although some *Sb*-specific modifications are necessary. This section will cover recent advances in our ability to perform transformations, integrate DNA into the genome, select active promoters, and secrete recombinant proteins using *Sb*. The focus will be on enabling technologies rather than specific applications, which are described in later sections.

### 2.1. Transformation

DNA transformation techniques developed for *Sc* are effective in *Sb* with minimal optimizations. Two transformation methods are commonly used for yeasts: lithium acetate/PEG/ssDNA and electroporation. The lithium acetate/PEG/ssDNA transformation method relies on cations to soften the cell wall and disrupt ionic interactions around the cell membrane, followed by a heat shock step to encourage DNA uptake [21]. Complexation of the DNA of interest with a single-stranded carrier (usually salmon sperm DNA) facilitates DNA entry. In 2014, Douradinha *et al*. compared the efficacy of this approach in *Sc* and *Sb* [22]. While transformation efficiencies were lower for *Sb*, the lithium acetate method succeeded with minimal optimizations, namely performing the growth steps at 37 °C instead of 30 °C [22]. In addition to using the higher growth temperature for *Sb*, Durmusoglu *et al*. also found that incubating the transformation at 42 °C for 1 h was the optimal time to generate successful transformants [18].

An additional transformation method commonly used for *Saccharomyces* yeasts is electroporation. Electroporation uses electrical current to disrupt the cell wall and membrane, promoting the entry of external DNA [23]. Extensive electroporation protocol optimization has been done for *Sc,* as shown in Benatuil *et al*. Some manipulated variables include cell density, cell conditioning agents, and components in the electroporation buffer [24,25]. Benatuil *et al*. showed that the two most important aspects of *Sc* electroporation were the cell conditioning steps with LiAc or DTT and the inclusion of sorbitol into the electroporation buffer. Without these pre-treatment steps, a large loss in transformation efficiency was observed, with an 88% decrease in efficiency without LiAc and a 94% decrease in efficiency without DTT. Similar to the results of excluding the pretreatment steps, when sorbitol was excluded from the electroporation buffer, a > 97% loss in transformation efficiency was observed [25]. With their optimized *Sc* electroporation method, Benatuil *et al*. observed transformation efficiencies as high as 1.3×10^8^ colonies per 1 μg of vector DNA [25]. To test electroporation efficiency for *Sb* specifically, Hamedi *et al*. used the *Sc* electroporation protocol developed by Benatuil *et al*., which only yielded several hundred *Sb* colonies per 1 μg of plasmid [26]. Ultimately, the results displayed by Douradinha *et al*., Durmusoglu *et al*., and Hamedi *et al*. show that transformation methods initially developed in *Sc* are transferable to *Sb*, albeit with varying efficacies.

### 2.2. Methods for selection of plasmid-containing strains

Similar strategies exist for the maintenance of plasmid transformants of *Sb* and *Sc*. Both the 2μ (high-copy episomal) and CEN/ARS (low-copy centromeric) origins of replication are commonly used in *Saccharomyces* yeast. Fortunately, *Sb* can replicate both 2μ and CEN/ARS-based plasmids, although copy number differences remain to be determined. Although *Sb* is susceptible to many of the same antifungal agents as *Sc*, and the same corresponding resistance genes have been used, such as *KanMX*, *ZeoR*, *NatR*, and *HygR,* it is undesirable to use antifungals *in vivo* as a selection marker, as these agents will disrupt the microbiome [18]. Instead, most studies harness engineered uracil, histidine, tryptophan, and leucine auxotrophies to maintain recombinant plasmids. Uracil auxotrophy was initially obtained via UV mutagenesis and selection for 5-fluoro orotic acid resistance [26,27]. Unfortunately, UV mutagenesis can lead to off-target mutations, as observed in both studies [26,27]. Wang *et al*. also created an uracil auxotroph in *Sb* using a Cre-Lox system, as previously validated in *Sc* [28]. The Cre-Lox system is more target-specific and has a lower probability of off-target mutations than UV mutagenesis. However, it is not a scarless editing technique, as it leaves behind loxP sites [28]. To overcome this issue, Liu *et al*. used CRISPR/Cas9-facilitated editing to inactivate *Sb*’s *URA3* (uracil), *TRP1* (tryptophan), *HIS3* (histidine), and *LEU2* (leucine) genes [29]. This study thus provided three additional *Sb* auxotrophic strains and validated the use of CRISPR-Cas9 to manipulate *Sb* [29]. This method is highly targeted, unlike UV mutagenesis, and scarless, unlike Cre-Lox. The Liu study successfully created four single auxotrophic strains and one double auxotrophic strain (*ura3/his3*); they also showed that tools and plasmids initially designed for *Sc* could be applied to *Sb* with a relatively high success rate. These foundational publications generated the first auxotrophic *Sb* strains that are still used today.

Despite these successes, the most appropriate strategy for plasmid maintenance *in vivo* remains to be determined. While genomic integration (described below) requires no selection, plasmids offer substantial advantages related to ease of use and high copy numbers. The nutrient-rich environment of the gut would seem to exclude the four auxotrophies mentioned above. However, Durmusoglu *et al*. observed no detectable loss of a plasmid encoding uracil prototrophy and β-carotene production over 14 days in the germ-free mouse gut [18]. Whether this reflects a true lack of uracil in the germ-free gut or a selection advantage for β-carotene production remains unclear. Nevertheless, future efforts should develop plasmid systems exploiting essential genes (perhaps similar to diaminopimelic acid auxotrophy in *E. coli* [30]) or metabolism of externally-provided carbon sources to ensure plasmid maintenance in the GI tract.

### 2.3. Genomic integration

For biotherapeutic engineering, genomic integration of DNA has become increasingly popular compared to plasmid-based expression. This is because integrating into the genome makes the inserted DNA more stable, less burdensome to the host cell, and does not require a selection scheme. Specific methods for genome editing in *Sb* largely mirror those used in *Sc,* with no reported differences between the two strains. While genomic integration is advantageous, this method benefits from well-characterized genomic integration sites, as different genomic regions enable different relative expression levels [31,32].

The earliest study performing genomic integration in *Sb* was performed by Douradinha *et al*., who used DNA flanked by δ sites to integrate exogenous DNA into these normally repetitive locations on the genome [22]. However, because *Sb* lacks many of the δ sites found in *Sc,* this system was found to be less than ideal for integration [22]. An additional integration method recently explored for *Sb* is the PiggyBac transposon system [33]. This transposon system uses PBase recognition sites to cut the expression cassette out of the plasmid and paste it into the *Sb* genome [33]. PiggyBac transposition could be a promising genome editing technique for *Sb*, as it does not cause genome instability due to chromosomal rearrangements and can integrate large DNA fragments containing multiple genes [33]. Other methods used to integrate exogenous DNA into the yeast genome include Cre-Lox and CRISPR [18,28,29]. As described above, the primary advantage to using CRISPR-based methods for genomic integration is that it is target-specific and scarless. Numerous neutral locations for stable chromosomal integration on different chromosomes, deemed integration loci, have been identified in *Sc* and characterized based on the expression level they enable [34,35]. Durmusoglu *et al*. also characterized the expression of two fluorescent reporters (mRuby2 and mTurquoise2) at five integration loci in *Sb* and measured gene expression at each site [18]. Expression varied across the five integration sites, but patterns of gene expression were similar between the two genes [18]. These observations, coupled with the large body of knowledge on integration sites in *Sc*, including portable toolkits such as the Yeast Toolkit for Modular, Multipart Assembly (YTK) [36] and Multiplex Yeast Toolkit [35], are likely to facilitate the generation of stable yeast therapeutics. Looking forward, the suitability of different genomic integration sites in divergent environmental conditions, including those in the intestine, should be investigated.

### 2.4. Promoter libraries

Promoter libraries are crucial to living therapeutic development as they allow for fine-tuning of gene expression, for example, to balance and maximize flux through a metabolic pathway or to maintain drug production within its therapeutic window. Douradinha *et al*. identified some well-characterized promoters in *Sc* with high homology to those in *Sb* [22]. However, this study did not characterize their expression. In 2021, Durmusoglu *et al*. characterized eighteen constitutive *Sc* promoters in *Sb*. For most tested promoters, expression levels in *Sb* and *Sc* were similar [18], except for *pALD6*, which was much stronger in *Sb* than in *Sc*. In 2024, Sands *et al*. analyzed twelve constitutive *Sb* promoters with high sequence homology to *Sc* and characterized their expression levels in *Sb* [37]. These promoters were tested in different conditions, including aerobic, anaerobic, differing carbon sources, and in the gastrointestinal tract. Sands *et al*. corroborated that promoter expression levels in *Sb* generally matched the expected pattern seen with *Sc* promoter expression. However, in several instances, deviations in expression occurred, such as expression levels depending on specific carbon sources [37]. Sands *et al*. observed that strains grown in fructose had a similar promoter expression profile to what was observed in glucose, while sucrose appeared to have an overall negative effect on promoter expression. In addition to sucrose and fructose, Sands *et al*. also tested inulin and acetate as carbon sources. The promoter expression profile for strains grown in acetate seemed to rely heavily on the presence of oxygen, while expression levels were generally elevated for the strains grown in inulin. To add to these constitutive promoters, in 2024, Durmusoglu, Al’Abri, and Haller *et al*. measured the activities of five inducible promoters [20]. These promoters responded to galactose, xylose, IPTG, copper, or anhydrotetracycline. All five promoters could tune expression based on inducer levels, but the promoters had variable levels of uninduced yeast expression (i.e., leakiness) and cross-talk levels. Excitingly, these inducible systems could be linked to form simple logic circuits, such as AND gates, which were active both *in vitro* and *in vivo*. These studies are important foundations for *Sb* engineering as they allow researchers to quickly select desirable promoters for their application. Looking forward, existing yeast toolkits such as the YTK [36] would benefit from augmentation with data on their performance across divergent yeast species, including *Sb*.

### 2.5. Secretion and surface display

The secretory pathway in *Saccharomyces* yeasts is complex, relying on many chaperone proteins to manipulate and transport nascent proteins to the cell surface and extracellular environment. As outlined by Meirong *et al*., current efforts to engineer secretion in yeasts focus on three main areas: secretion signal optimization, endoplasmic reticulum (ER) folding, and vesicle transport [38]. Secretion signals are short, N-terminal peptides that assist with protein translocation into the ER and post-ER sorting. Once in the ER, many chaperone proteins facilitate modifications, including folding and glycosylation. After leaving the ER, vesicles transport modified proteins to the Golgi and, after further modification and signal sequence cleavage, towards the cell wall for exocytosis.

Engineering the signal sequence has become a popular strategy for modulating protein secretion rates, as it requires altering protein sequences rather than extensive strain engineering. The signal sequence consists of a pre-sequence, which determines the translocation method nascent proteins use to enter the ER, and a pro-sequence, which governs sorting upon exit from the *trans*-Golgi network [39]. The *Sc* ɑ-mating factor is a common choice for preliminary secretion optimization, as it is efficacious for various proteins (Table 1). Recent advancements have explored more native, synthetic, and hybrid signal sequences to further optimize secretion. However, predicting the ideal signal sequence for a given protein remains challenging, as no clear relationship exists between sequence properties and secretion efficiency [40]. Notably, studies like those from Xue *et al*. have identified key features of native *Sc* signal sequences, including distinct N-, H-, and C-regions, each contributing specific characteristics such as hydrophobicity and charge balance to enhance ER translocation and signal peptidase efficiency [41].

Synthetic signal sequences offer further opportunities to optimize secretion by addressing challenges like improper sequence cleavage, vesicle trafficking errors, and metabolic burden. Durmusoglu *et al*. demonstrated that optimizing signal sequences in *Sb* could significantly improve secretion of 8-mer peptides, as seen with hybrid sequences like preOST1-proαMF, which achieved secretion rates of 463 mg/L, as compared to 255 mg/L with the commonly chosen α-mating factor [42]. These findings underscore the potential of hybrid signal sequences to enhance secretion while balancing metabolic stress. By leveraging a combination of native and synthetic elements, researchers aim to fine-tune the secretory pathway, increasing yields without requiring extensive modifications to the host strain’s genome.

In addition, genome editing has been employed in tandem with secretion signal sequence optimization to increase protein secretion in *Sc* and *Sb*. While more labor-intensive than secretion signal engineering alone, genome editing may provide more broadly acting improvements to the yeast secretion pathway. Recent work by Durmusoglu *et al*. showed that targeted gene knockouts against secretion pathway elements affect recombinant peptide secretion rates in *Sb*. Targeted genes in this study related to lipid composition, ER-associated degradation (ERAD), Golgi modification and trafficking, and protein degradation. Knockout of vacuolar and extracellular protein degradation genes showed the most significant increase in secretion rates, enabling over 2000 mg/L recombinant peptide to be secreted, compared to the 500 mg/L titer in wild-type *Sb* [42]. Combinations of these knockouts were also shown to be effective in increasing secretion rates, as shown by the mutant *SbΔpep*4Δ*prb*1Δ*yps*1Δ*ape1*, which enabled a peptide secretion titer of 5045 mg/L [42]. While promising, these mutant strains were not tested with larger proteins, and it is unclear if these genetic targets are effective for producing proteins with divergent biochemical properties.

Yeast surface display also shows promise for living therapeutics. To date, two methods of achieving surface display have been demonstrated. In the earliest of these, Wang *et al*. used the traditional a-agglutinin-based display system to display eGFP and EtMIC2 [43]. More recently, Heavey *et al*. used a similar system, expressing the Aga1 anchor and an Aga2 fusion protein with a terminal monomeric streptavidin. The authors then conjugated the Aga2 fusion with biotinylated antibodies against Extracellular Matrix (ECM) proteins before administration to mice in an acute colitis model [44]. While the antibodies used were not recombinantly produced by *Sb*, this design provides a promising alternative for displaying difficult-to-express proteins before oral administration. While *Sb*-based display systems are still in their infancy, these early successes show the feasibility of a functionalized probiotic yeast surface display platform for treating and detecting gastrointestinal diseases.

Recently developed toolkits have significantly increased the speed at which yeast expression and secretion can be studied [36,40]. Unfortunately, no known correlation exists between the physical properties of a recombinant protein and the choice of secretion signal that would best optimize its secretion [40]. Generally, methods for enhancing *Sb*’s secretion and display titers generally follow the same strategies employed in *Sc*. However, the substantial phenotypic differences between the two strains, including the larger cell wall size in *Sb* [17], *Sb*’s reported ability to secrete unique proteases [10,42], and the uniqueness of the gut environment indicate that some *Sb*-specific optimizations likely exist. Identifying such factors will require new methods for high-throughput testing of protein secretion/display in gut-like environments.

## 3. Load-enhancing factors

The “load” of a host-associated microbe refers to its viable population at a particular time on its host. Within the gut, probiotics must compete for physical space and availability of resources while at the same time resisting killing by viruses and the host immune system. *Sb* is an attractive target for load enhancement due to its natural probiotic features. Engineered versions of *Sb* may exhibit load-dependent benefits, as greater numbers of producing cells are likely to produce larger drug titers. Thus, significant effort has been expended to understand the determinants of *Sb*’s load in the gut and to identify strategies to modulate this load.

### 3.1. Wild-type probiotic load studies

Blehaut *et al*. investigated how long *Sb* takes to reach a steady state concentration within the gut of humans, rats, and mice using single-dose or continuous administration [45]. A significant conclusion from the authors was that *Sb* takes approximately three days to reach a steady state concentration within the gut in humans if the probiotic is administered continuously. After stopping the administration of *Sb*, clearance occurs within three to five days. This study was critical for showing that *Sb* is not a long-term colonizer of the human gut.

**Fig. 1.**
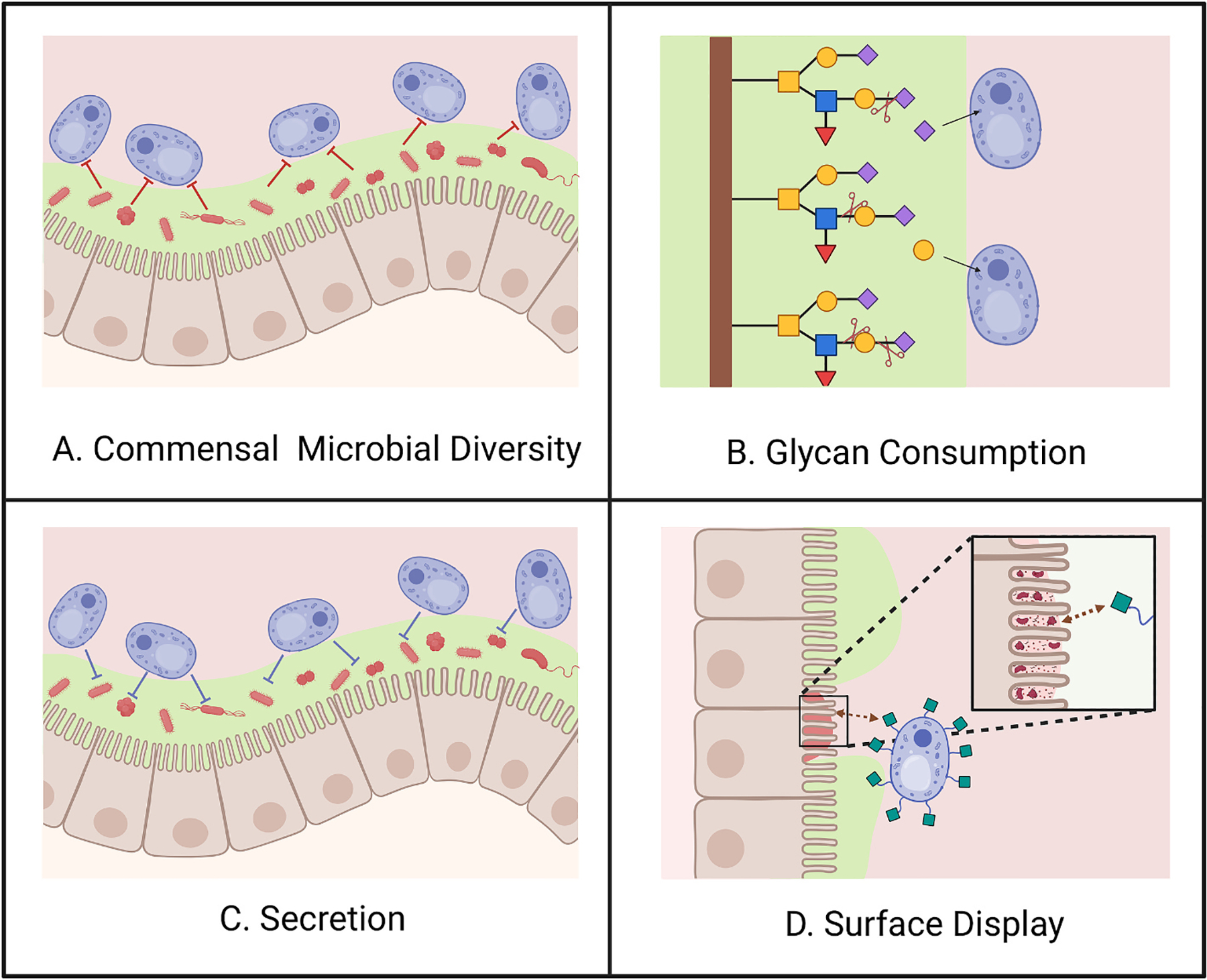
Factors affecting probiotic load in the gut. The figure above illustrates the various factors that can lead to an increase or decrease in probiotic load of engineered *Sb* (blue). **A. Commensal microbial diversity:** Commensal microbes (red) residing in the gut mucus (green) can outcompete *Sb* for carbon sources. **B. Glycan consumption**: Engineering *Sb* to consume mucin glycans present in the gut mucus can give it a competitive advantage and allow it to reside in the gut for longer periods. **C. Secretion:** Secretion of molecules like acetic acid can lead to an inhibitory effect on the microbes in the gut (red) which reduces their ability to compete with *Sb*. **D. Surface Display:** Surface display of molecules that bind to extracellular matrix proteins of inflamed intestinal epithelial cells allows *Sb* to perform its therapeutic effect at the site of action.

In 2021, Durmusoglu and Al’Abri *et al*. analyzed the residence time of *Sb* in mice to determine whether antibiotic administration could affect the yeast’s colonization profile [18]. The authors first administered 10^8^ colony-forming units (CFUs) of *Sb* into germ-free mice, which are devoid of a gut microbiome. They observed that *Sb* resided in the gut for over 30 days without noticeable harm to the mice, indicating that the gut is, in principle, supportive of long-term yeast survival. They then measured the residence time of *Sb* in conventional mice containing an intact microbiome or one affected by two different antibiotic regimens. Treatment 1 consisted of 1 mg/mL penicillin and 2 mg/mL streptomycin in water four days before beginning *Sb* administration, with continued antibiotic supplementation throughout the time course of the experiment. Treatment 2 followed the same dosage, but stopped supplementation after the first gavage of *Sb* [18]. The authors found that continued antibiotic treatment prolonged the residence time of *Sb* within the gut, indicating that gut bacteria significantly limit the ability of *Sb* to thrive in this environment (Fig. 1A).

A subsequent study conducted by Hedin *et al*. [46] further showed that an antibiotic cocktail composed of broad-spectrum antibiotics like ampicillin, kanamycin, metronidazole and vancomycin improved the colonization of *Sb* by 10,000-fold as compared to nonantibiotic-treated mice. In this study, one cohort of mice were supplemented with antibiotics, while the other group received no antibiotics. The researchers observed that the mice treated with the broad-spectrum antibiotic cocktail resisted *Sb* washout for longer, showing detectable quantities of *Sb* in fecal matter up to ten days, as compared to the two-day washout of the non-antibiotic-treated mice. These studies highlight the utility of antibiotics to enhance *Sb* load during preclinical testing, and the potential ability of *Sb* to colonize the gut more readily if a patient is already taking antibiotics to treat a bacterial infection.

While the studies above showcase that antibiotic administration could potentially improve colonization load, antibiotics should not be used solely for this purpose as they can disrupt the microbiome and select for antibiotic-resistant bacteria [47,48]. The following section will showcase the means and methods undertaken by research groups to sustainably improve the colonization load of *Sb* without antibiotics.

### 3.2. Probiotic load enhancement studies

Scientists have attempted to improve the colonization of *Sb* by adopting similar strategies to those used by long-term colonizers of the gut like *Akkermansia muciniphila* and *Bacteroides thetaiotaomicron* [49]. These efforts fall into three main categories: mucus consumption, competitor clearance, and adherence to gut mucus (Fig. 1 B–D). Gut mucus is a heavily glycosylated protein that serves as a carbon source for many gut bacteria. Despite its complexity, mucus glycans comprise only five major monosaccharides: galactose, fucose, N-acetylglucosamine, N-acetylgalactosamine, and sialic acid. Liu *et al*. explored why *Sb* cannot grow well on galactose. The results showed that the gene *PGM2*, which codes for phosphoglucomutase, contains a point mutation that introduces a premature stop codon, preventing *Sb* from metabolizing galactose efficiently [13]. The authors then repaired the point mutation in *Sb* using the same sequence of the *PGM2* gene in *Sc*, leading to much better growth of *Sb* on galactose. Durmusoglu, Al’Abri, and Haller *et al*. tested the load of this *Sb* strain with the repaired *PGM2* gene (*SbGal*^+^) versus wild-type *Sb* in antibiotic-treated conventional mice [20]. The authors found that this strain did not exhibit increased load alone. However, dietary galactose supplementation enhanced the load of the *SbGal*^+^ strain by more than three orders of magnitude versus the wild-type strain. These results showed that the low availability of free galactose in the gut (due to lack of hydrolysis from mucus glycans or fast catabolism by gut bacteria) may be a barrier to colonization.

Kim *et al*. engineered *Sb* to consume L-fucose as a sole carbon source. The authors overexpressed a native fucose transporter from *Sc* and coding sequences of L-fucose metabolism genes from *Escherichia coli* [50]. *Sb* was transformed with plasmids containing the fucose-consuming genes, and its growth was tested on aerobic, microaerobic, and anaerobic conditions. The authors concluded that the best growth was observed when *Sb* was grown in aerobic conditions, as the metabolic flux was directed through the (S)-lactaldehyde-(S)-lactate-pyruvate pathway, which is highly oxygen dependent [50]. The results of this study showed that although it is possible to engineer *Sb* to consume fucose, the oxygen dependency of the pathway may be a roadblock to prolonged colonization.

To enable *Sb* to inhibit the growth of competitor bacteria, de Carvalho *et al*. identified the mutations in *Sb* that cause high acetic acid accumulation and used these to engineer a strain of *Sb* that can tolerate and produce higher concentrations of acetic acid [51]. Acetic acid is an important substrate in the gut; it is used in the production of butyrate and assists in lowering the pH in the lumen, which is one of the body’s natural defenses against pathogens. De Carvalho *et al*. sought to increase acetic acid production, hypothesizing that higher environmental concentrations of acetic acid would bolster defense against pathogens. The authors achieved this via homozygous deletion of *ACH1* and over-expression of *ALD4*. They further demonstrated antimicrobial efficacy *in vitro* against known gut pathogens like *Klebsiella pneumoniae*, *E. coli*, and *Enterobacter* strains in aerobic and anaerobic conditions, which may imply that this antimicrobial effect may be observed in gut-like environments as well. In mice, it was observed that their engineered strain persisted for longer in the gut, potentially by decreasing the abundance of bacterial competitors. These results were obtained when the cells were grown using glucose as a carbon source. Because glucose concentrations in the gut vary substantially between meals, the authors mention that a strain of *Sb* that can produce acetic acid under high and low glucose concentrations would be ideal. While the authors claimed that their ENT3 strain produces the highest amount of acetic acid under both high and low glucose concentrations, this quantification was conducted in an aerobic environment. Therefore, additional studies are needed to test the efficacy of their strain in mouse models.

To allow *Sb* to adhere to the epithelium and resist washout by intestinal peristalsis, Heavey *et al*. [44] harnessed surface display [43] to improve its colonization specifically in ulcerative colitis mouse models. The authors displayed antibodies that bound Extracellular Matrix (ECM) proteins, namely fibronectin, fibrinogen, and collagen IV. These ECM proteins are deposited in colonic tissue during disease. The authors observed that *Sb* targeting fibronectin had the longest residence time in an acute Dextran Sodium Sulfate (DSS)-induced murine model of colitis. Furthermore, *Sb* targeting collagen IV was shown to have the highest probiotic cell concentration and reduced inflammation during chronic DSS treatment. This platform provides great promise for developing target-specific engineered probiotics. However, the authors note the need for patient-specific and lesion-specific targeting ligands as the ECM dynamically remodels in response to disease progression and severity of lesion inflammation [44]. Nevertheless, this methodology holds potential for programmable yeast colonization through surface display.

While improving the probiotic’s load may provide therapeutic benefits, this strategy must be performed cautiously and be accompanied by monitoring disruptions in the commensal microbiome. Typically, an individual’s gut microbiome is stable and provides colonization resistance against pathogens through the secretion of antimicrobials, inhibitory metabolites, or by consuming key nutrients [52]. Introducing *Sb* as a permanent colonizer may lead to changes in the gut microbiome composition, and these effects may lead to susceptibility to infections. Furthermore, the genetic features distinguishing benign colonization and infection are not fully elucidated. If an engineered strain of *Sb* outcompetes the commensal microbiome, it may lead to dysbiosis [53]. Hence, care must be taken to improve *Sb*’s colonization profile while maintaining the gut microbiome’s integrity. Another factor to consider is the ability of yeast to produce ethanol from fermentable carbon sources like glucose. Production of ethanol may be a side-effect to enhanced colonization time in the gut and long-term studies in animal models may need to take this into account to truly determine if higher probiotic load through increased colonization time is beneficial to the host. Studying the colonization of *Sb* and its interactions with native gut members, as well as engineering *Sb* to safely increase its gut load without side effects, would greatly benefit biotherapeutic development and may illuminate mechanisms by which native gut commensals achieve their long-term residence within the gut.

## 4. Sensors

**Fig. 2.**
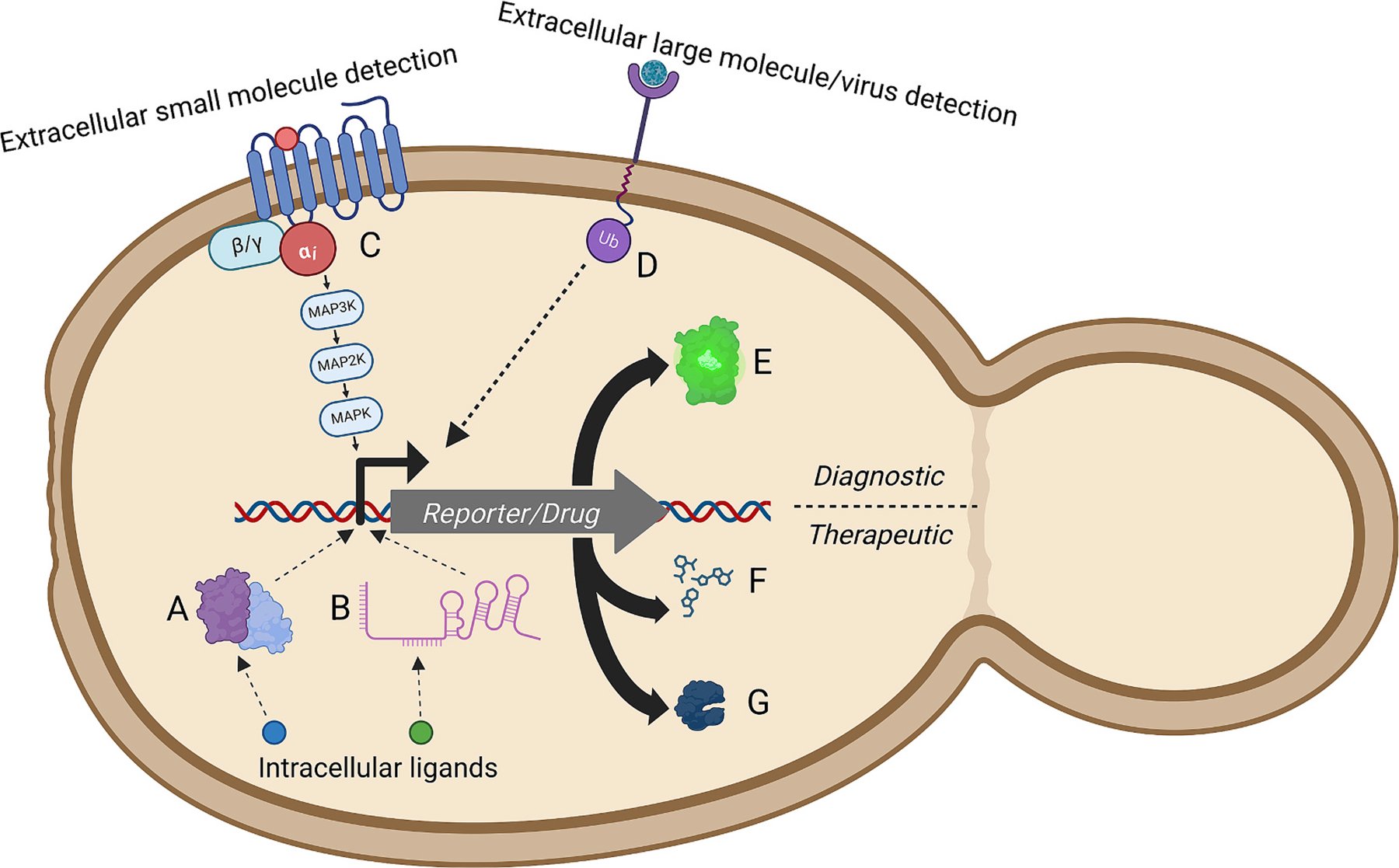
Modalities of biosensing in probiotic yeast. Several approaches to date have been used to enable biomarker-responsive behavior in probiotic yeast. **A. Ligand-responsive Transcription Factors** detect intracellular ligands via a conformational change, thereby repressing/derepressing a cognate promoter. **B. RNA-based** sensors exploit ligand-responsive RNA structure to detect the presence of intracellular molecules. **C. G Protein-Coupled Receptors** detect extracellular ligands and activate MAPK signaling through a corresponding G-Coupled protein, best used to detect small molecules. **D. Stem Receptor** fuses native cell wall stress sensors to link a ubiquitin-activated intracellular expression to an extracellular receptor, best used to detect large molecules and viruses. **E. Fluorescent Reporter** used to assess sensing mechanism engineering, or for diagnostic purposes. The reporting mechanism can also be engineered to produce a **F. small molecule or G. protein** in response to a detected ligand, the ultimate application of biosensors to produce live biotherapeutics.

The microbial composition of the gut varies with a variety of human diseases. In fact, it is rare to find diseases for which the gut microbiome does not change. Regardless of the direction of these associations, an altered microbial community implies changes to gut metabolites that may serve as biomarkers for disease. The ability of a probiotic to sense these biomarkers would be useful for several reasons, including early diagnosis of a disease and rapid initiation of drug production in response to disease. Here, we review sensing modalities used in living yeast therapeutics, including intracellular ligand-responsive Transcription Factors (TF) (Fig. 2A) and RNA-based sensors (Fig. 2B), as well as extracellular G Protein-Coupled Receptors (GPCRs) (Fig. 2C) and stem receptors (Fig. 2D). We also discuss the therapeutic applications of these biosensors’ reporting mechanisms, both for diagnostics (Fig. 2E) and drug delivery (Fig. 2F–G).

### 4.1. Intracellular biosensing modalities

Often, small molecules from the cell exterior can enter the cell via passive diffusion, making their intracellular levels faithful reporters of extracellular conditions. In addition, the detection of small molecules or proteins produced within the cell itself may be of interest. The most widely used biosensors to detect intracellular ligands are Transcription Factor (TF)-based, which undergo a conformational change when an analyte binds, subsequently causing binding/unbinding to a promoter sequence, and repressing or de-repressing transcription. Historically, heterologous and endogenous TF/promoter pairs from both prokaryotes and eukaryotes have been used. TF activity may directly control the expression of a reporter gene, or it may control the expression of another TF, for example when assembling multiple biosensors for logical computations [54].

Engineered transcription factors can be broken into two key components: the DNA-Binding Domain (DBD) and the Effector-Binding Domain (EBD) [55]. The EBD serves as a sensing mechanism that can be sourced from nature and/or can be engineered through rational protein engineering or directed evolution if no suitable natural sensor is present [55,56]. For instance, D’Ambrosio et. al imported a TF system from the bacterium *C. crescentus* into an *Sc* platform to build a vanillin detector, suggesting bacteria may be used as a source of EBDs [55]. However, natural EBDs can also be subject to feedback inhibition or transcriptional crosstalk. These can be addressed by knocking out interfering non-essential pathways, as well as expressing the transcription factor components with non-native promoters [56]. Although the existence of suitable EBDs for arbitrary intracellular ligands is not guaranteed, advances in computational protein design and high-throughput screening are constantly expanding the set of molecules that can be coupled to gene expression [57].

Dacquay *et al*. used the yeast TF Yap1p to develop an *Sb* strain that is responsive to the presence of reactive oxygen species [58]. This study built upon earlier work by Zhang *et al*., who developed a biosensor to monitor the redox state of *Sc* by expressing a fluorescent reporter under control of a modified version of *pTRX2*, the target of Yap1p [59]. Yap1p helps transcribe several genes that combat oxidative stress in yeast and is activated in response to stress. Zhang *et al*. modified *pTRX2* by adding additional Yap1p binding sites to create a promoter that responded proportionally to levels of NADPH in the cell [59]. One consequence of these efforts was that the length of *pTRX2* was greatly increased, potentially reducing cloning efficiency. Therefore, Dacquay *et al*. expanded upon this concept and engineered a shorter *pTRX2* variant [58]. To do so, they altered the space between Yap1p binding sites, modifying the copy number of *YAP1*, altering the binding site sequence, and modifying the 5′UTR [58]. Dacquay *et al*. also implemented a positive feedback system to recover the same expression of the mCherry reporter that was achieved with the longer promoter. Finally, the authors moved this system into *Sb*, thus generating a strain of probiotic yeast that could be used to detect oxidative stress, for example during inflammation of the gut.

As mentioned in Section 2.4, Durmusoglu, Al’Abri, and Haller, *et al*. implemented TF-based biosensors of galactose, xylose, copper, IPTG, and aTc to develop small-molecule-responsive logic circuits that were operational *in vivo* [20]. While the focus of this study was the detection of compounds provided in the diet, the authors also observed a significant level of activation from the galactose-responsive promoter *pGAL1* even in the absence of galactose in the drinking water. Based on the well-known induction requirements of the *pGAL1* promoter in *Sc* [60], this result potentially indicates the presence of free galactose in the mouse gut, in tandem with low free glucose concentrations.

The plethora of TF-based biosensors available in prokaryotes, as well as the diversity of TF-based biosensors already found in and/or developed for *Sc*, indicates that TF-based biosensing is a ripe area for further development of “smart” biosensing probiotic yeast. Their modularity and simplicity make engineering these systems straightforward, making these a promising approach provided that the metabolite of interest is able to enter the yeast nucleus where the TF is located.

Another approach for detecting intracellular metabolites in *Sb* uses RNA and enables additional levels of transcriptional regulation using orthogonal transcriptional modulation, or transactivation [61]. First, a synthetic promoter comprising 20 random DNA bases not found in the *Sb* genome serves as the targeting sequence for the 5′ end of a scaffold RNA (scRNA). In addition to this target sequence, scRNA contains two hairpins: one that binds to dCas9 and one that binds to a specific transcriptional activator. This system therefore requires two keys to unlock reporter transcription: the targeting sequence on the scRNA and the transcriptional activator, which can be inducibly expressed. Kwak *et al*. built scRNA molecules with different targeting sequences and demonstrated orthogonal GFP reporter expression [61]. This *Sb*-based platform is another option for probiotic strain development, and has been implemented to report viral detection, as described below.

### 4.2. Extracellular biosensing modalities

While intracellular chemical signals are naturally produced in many therapeutically relevant scenarios, biosensing platforms often have to be engineered to transmit extracellular chemical changes to the internal biosensing platform. The most well-characterized and engineered of these extracellular biosensors are G Protein-Coupled Receptors (GPCRs). GPCRs are 7-transmembrane proteins linked to Mitogen-Activated Protein Kinase (MAPK) signaling pathways, which make up an expansive and varied class of receptors in eukaryotes. Extracellular ligands alter GPCR conformation upon binding, in turn dissociating the Gα subunit from the remaining G*β*-G*γ* dimer. This dissociation activates the MAPK pathway, which can subsequently activate hundreds of downstream genes. *Sc* harbors two native GPCR pathways: one for mating pheromones and one for sugar. The pheromone-responsive pathway is often used as a platform to express heterologous GPCRs, and many human-derived GPCRs have been reconstituted in yeast via this system [62,63].

Shaw *et al*. “refactored” *Sc*’s GPCR signaling pathway to better correlate ligand interactions with reporter response by deleting non-essential endogenous genes and tuning the response using promoter libraries [56]. Ideally, ligand dosage in the extracellular matrix should correlate with the reporter output in an engineered strain. Native negative feedback response loops are responsible for obscuring this correlation, so Shaw *et al*. removed the gene encoding a negative regulator, *SST2*. Furthermore, they demonstrated that the G protein subunits and GPCR-ligand binding could be modified depending on the desired dose–response curve. They demonstrated this using human adenosine (*A2BR*) and melatonin (*MTNR2A*) GPCRs, which detect essential gut neurotransmitters.

Jensen *et al*. built upon the Shaw platform and developed the first ligand-sensing strain using GPCRs in *Sb*. *Sb* is a MATa/α diploid and, as mentioned above, is unable to sporulate. This poses a challenge for GPCR signaling, which requires yeast of a single mating type. Jensen *et al*. used CRISPR/Cas9 to generate a homozygous MATa/a diploid *Sb* strain, thereby re-sensitizing it to the presence of α factor [64]. In this engineered homozygous strain, they knocked out *SST2* and separately expressed heterologous adenosine, melatonin, serotonin, and Mam2 (sensitive to the *S. pombe* mating factor) GPCRs [64]. For each GPCR, Jensen *et al*. also introduced chimeric Gα subunits to assist signaling to the native MAPK pathway [64]. Using a GFP reporter, they demonstrated that receptor-G protein coupling strength could be assessed independent of the ligand, which will facilitate GPCR engineering for other ligands going forward. The immediate significance of this study is the development of an *Sb* platform that not only can successfully detect ligands using GPCRs, but also tune the strength of the reporter output.

Scott *et al*. engineered *Sc* to sense extracellular ATP (eATP), a biomarker of inflammatory bowel disease [65]. To do this, the authors integrated the human P2Y2 purinergic receptor into the *Sc* genome. Because the wild-type receptor detects both eATP and eUTP, the authors employed directed evolution to yield a mutant P2Y2 receptor specific for eATP. The most specific variant was then used to drive the expression of therapeutic apyrases from wheat and potato [65]. Apyrase cleaves ATP into adenosine, which has anti-inflammatory properties. The authors then showed that when delivered to a mouse model of inflammatory bowel disease, only the yeast strain with both the biosensor and the apyrase expression was able to efficiently mitigate disease [65]. While p-values between the constitutive and inducible engineered strains were not reported, the inducible strain showed a qualitatively better response relative to constitutive control, suggesting inducible apyrase expression is better than constitutive expression for therapeutic applications. This is the first study demonstrating the coupling of biosensor activation to drug delivery in yeast, although it is with an *Sc* strain. The authors note that there may be certain advantages to maintaining such a system in *Sc*, for example when transience of the therapeutic is desired [65].

Several other studies report the use of GPCRs to generate therapeutically-relevant yeast biosensors. Ostrov *et al*. developed an *Sc* biosensor that produced carotenoid pigment in response to detecting *C. albicans*, a fungal human pathogen [66]. They introduced a lycopene synthase pathway, whose final gene, *CRT1*, was expressed under control of pheromone-inducible *pFUS1*. With this reporter, they were able to sensitively and specifically detect the human pathogens *C. glabrata*, *L. elongisporus*, and *P. brasiliensis* [66]. This platform could therefore be developed either as an *in vivo* or *in vitro* diagnostic biosensor, depending on the reporting mechanism used. Lengger *et al*. demonstrated dose-responsive serotonin detection at gut-relevant pH levels. Transferring these systems to *Sb* could enable development of diagnostics and gut-targeting live yeast therapeutics [67].

Taken together, by virtue of their ability to detect extracellular ligands (i.e. when intracellular concentrations do not reflect extracellular concentrations), GPCRs comprise a powerful and expanding biosensing toolkit for the probiotic yeast engineer. Future challenges in this space include the inability to easily “drop in” most GPCRs to a yeast biosensing application – some activity optimization is usually required [56]. Furthermore, the placement of GPCRs within the cell membrane means that the cell wall (exterior to the membrane) may still pose a barrier to ligand binding, making these unsuitable for detection of, for example, very large molecules by probiotic yeast. To resolve this issue, Kwak, Dantas, and Virgin have disclosed a patent [68] that leverages stress sensors in the cell wall integrity pathway to build an *Sb* biosensor to detect viral particles, which are too large for GPCRs.

The cell wall integrity pathway includes sensor proteins that span the yeast cell membrane and wall. Kwak *et al*. fused the cell wall stress sensor, Mid2p, to the transmembrane stress sensor, Wsc1p, to create a “stem” from the extracellular environment to the RNA-based transactivation reporting system described previously. A binding agent linked to the stem is displayed on the surface of the cell wall, and initiates reporter expression by releasing ubiquitin intracellularly [68]. The authors demonstrate the detection of *S. aureus* antigens using this system with GFP and mCherry. They also build a sensor for norovirus, a leading cause of gastroenteritis. They express CLM-1 as a binding agent in *Sb*, and demonstrate reduction of norovirus titer over 8 days in mouse experiments [68], presumably by preventing norovirus from accessing human cells. This platform can be used for sensors for other molecules too large for GPCRs, and link them to activation of a therapeutic for future probiotic application.

Most of the work on *Sb* biosensors has focused on improving the specificity and sensitivity of sensing mechanisms using fluorescent or colorimetric reporter systems, and the majority of such systems are used for diagnostics, not as live therapeutics. However, these same approaches, when used to trigger drug release in the gut, may enable the creation of strains that can autonomously monitor the gut for signs of disease, and initiate treatment at low biomarker concentrations, potentially even before an individual experiences disease symptoms. Looking forward, in addition to their roles as triggers for drug synthesis, these sensors may also be used to halt drug production, for example to maintain drug concentrations within a therapeutic window or to halt drug production outside of the gut environment.

## 5. Actuators and delivery

Colonization and biosensing are both essential “enabling technologies” for engineering a live microbial therapeutic. However, ensuring the organism can produce and deliver the biotherapeutic of interest in biologically relevant amounts is also critical. Table 1 highlights efforts made towards the secretion and surface display of recombinant biomolecules from *Sb*.

The first study reporting the engineering of *Sb* to secrete a recombinant molecule was published in 2013, in which *Sb* was engineered to mitigate inflammation caused by colitis by secreting interleukin-10 (IL-10) using the *Sc* α-mating factor [69]. While secreted IL-10 was successfully detected, reaching concentrations of up to 190 ng/mL in a colitis mouse model, there was no significant decrease in inflammation, indicating that either productivity or localization of the yeast was insufficient [69]. In 2016, Liu *et al*. transformed *Sb* to create three new strains expressing different heterologous proteins: lacZ, the xylose assimilation pathway, and human lysozyme [29]. In all three cases, Liu *et al*. showed that *Sb* successfully created and secreted these different proteins *in vitro* [29]. In the case of human lysozyme, the authors chose the chicken lysozyme secretion signal sequence, which resulted in higher secretion titers. These studies show that *Sb* can be genetically manipulated to produce and secrete heterologous proteins *in vitro*, using both native and non-native secretion signals. Shortly following this discovery, Bagherpour *et al*. successfully engineered *Sb* to produce an Ovalbumin-CPE fusion protein using the α-mating factor secretion signal [70]. The associated mouse studies showed promising results, as the mice fed Ovalbumin-CPE-producing *Sb* had elevated levels of IgG in the serum and IgA in fecal samples [70].

After this foundational work, recent years have shown a significant increase in research to genetically modify *Sb* in ways that make it a valuable biotherapeutic or a better probiotic. These studies focus on engineering *Sb* to secrete molecules in one of three categories: host-targeted molecules, non-targeted antimicrobials, or targeted antimicrobials.

### 5.1. Production of host-targeted compounds

Several studies investigated the ability of *Sb* to produce compounds that regulate the activities of its human host, using undigested dietary material as a substrate. One major type of these compounds is small molecules. In 2021, Durmusoglu *et al*. expressed four genes responsible for the biosynthesis of β-carotene, a vitamin A precursor, in *Sb* [18]. When tested in a mouse model, the strains were still functional and could produce relevant quantities of β-carotene [18]. An additional study in the same year by Jin *et al*. focused on engineering *Sb* to produce neoagrooligosachharides (NAOSs) by expressing an *endo*-type β-agarase. NAOSs are short fragments of agarose, a component of red macroalgae, and have been shown to have prebiotic effects in humans and can help mitigate inflammatory response pathways [74]. Unlike many other studies, multiple secretion signal peptides were tested, including the α-mating factor, chicken lysozyme, *Sta1*, and *Sed1* secretion signals. After determining that secretion was optimal using the *Sed1* secretion signal, Jin *et al*. successfully engineered *Sb* with the *endo*-type β-agarase that could hydrolyze agarose and secrete neoagarotetraose, a type of NAOS, into the supernatant [74]. In a final study, Tang *et al*. engineered *Sb* to produce L-Ergothioneine (EGT). EGT is a natural and nontoxic antioxidant that shows promise in protecting against stress-induced sleep and neuronal injury caused by amyloid β [33]. Using PiggyBac Transposition, Tang *et al*. successfully integrated the EGT biosynthetic pathway to generate and secrete EGT *in vitro* [33]. While these studies show that *Sb* can be engineered to produce beneficial small molecules in controlled *in vitro* settings, these strains would need to be tested in *in vivo* conditions to prove that their efficacy could continue to work in a more complex living system.

In addition to secreting small-molecule dietary supplements, additional studies have been conducted to secrete host-modulating proteins. In Liu *et al*., researchers secreted proteins to fight inflammation caused by Inflammatory Bowel Disease (IBD), as current treatments for IBD are unsuitable for long-term usage [72]. The first three proteins explored, IL-10, TNFR1-ECD, and alkaline phosphatase, showed no therapeutic effect in mice. However, the fourth candidate, Atrial Natriuretic Peptide (ANP), demonstrated significant improvement in disease activity index, body weight maintenance, and decreased expression of pro-inflammatory cytokines [72]. Using *Sb* to deliver ANP to IBD patients with colitis symptoms could be a promising treatment alternative to the current anti-inflammatory small molecules. Hedin *et al*. engineered *Sb* to produce Exendin-4 to combat diet-induced obesity [19]. Exendin-4 is a GLP1R agonist that has shown promise in treating obesity, but treatment is non-optimal, as it requires injections twice daily. Hedin *et al*. solved this inconvenient treatment strategy by integrating an Exendin-4 coding sequence into the *Sb* genome [19]. The engineered *Sb* showed no significant activity in reducing the obesity of mice at room temperature. However, treatment with Exendin-4-producing *Sb* and subsequent cold exposure resulted in a loss of body weight, decreased respiratory exchange ratio, and decreased small intestine gut content mass [76]. In another study, Yamchi *et al*. engineered *Sb* to express Polypeptide-P, a plant protein that decreases blood sugar [79]. *Momordica charantia* seeds are not ideal for Polypeptide-P production in treatments, as they have dilute active ingredients and their metabolites are toxic to humans [79]. Yamchi *et al*. successfully engineered *Sb* to secrete Polypeptide-P using the α-mating factor secretion signal and verified Polypeptide-P production using HPLC analysis. When the engineered *Sb* strain was administered to diabetic rats, the probiotic significantly lowered the blood sugar in the test animals [79].

For many compounds, presence in the bloodstream is a prerequisite for therapeutic activity, especially for compounds whose target is not in the gut. Gelli *et al*. engineered Sb to make and secrete one of four unique Cell-Penetrating Peptides (CPPs) to address this issue. CPPs are small peptides that help weaken the cell membrane and enable macromolecules to cross through, such as between the gut and the bloodstream [80]. This is useful for biotherapeutics, as many relevant molecules have difficulty delivering their biotherapeutic payload into host cells. Gelli *et al*. integrated a CPP into the *Sb* genome and tested the peptide’s ability to weaken the cell membrane *in vitro* using the Transepithelial Electrical Resistance (TEER) of Caco-2 monolayers [80]. In this assay, three of the four peptides successfully weakened the membrane, as shown by decreased TEER in the monolayers [80]. After weakening the cell membrane, FITC-dextran, a large macromolecule that generally cannot cross the cell membrane, was added to the monolayers. As the cells recovered, researchers observed an increase in the concentration of FITC-dextran passing across the cell membrane in samples containing CPPs [80]. Researchers then orally gavaged mice with the CPP-producing *Sb* strain followed by gavage of the FITC-dextran. Only one of the four peptides significantly affected the macromolecule’s translocation into the blood [80]. This study shows promise in using CPPs as an enabling technology to help deliver therapeutics into cells, which is a bottleneck in current biotherapeutic research. Overall, Durmusoglu *et al*., Jin *et al*., Hedin *et al*., Yamchi *et al*., Tang *et al*., and Gelli *et al*. demonstrate the potential of engineering *Sb* to create compounds that improve upon *Sb*’s native health benefits.

### 5.2. Production of non-targeted antimicrobials

In addition to engineering *Sb* to deliver molecules that aid in the general health of an individual, researchers have also used *Sb* to make biotherapeutic molecules that can target general pathogens. Baldera-Aguayo *et al*. engineered *Sb* to biosynthesize tetracycline analogs, specifically the fungal anhydrotetracycline TAN-1612 [75]. Tetracyclines and their analogs target a wide range of bacteria by binding to the 30S ribosomal subunit and preventing translation [75]. *Sb* is an attractive host for production of tetracycline analogs since yeasts are not susceptible to phages, like bacterial platforms more commonly engineered for tetracycline production. One engineered *Sb* strain from the library generated in this study produced TAN-1612 at 6.1 mg/L, suggesting *Sb* may be a viable host for antibiotic synthesis [75].

A second example of *Sb* producing an antibacterial compound is shown in Kim *et al*. [77]. In this study, researchers integrated a human lysozyme cassette into two locations on the *Sb* genome to increase lysozyme secretion titers. As with the previous study by Liu *et al*., the chicken lysozyme secretion signal was used to promote recombinant lysozyme secretion. The engineered *Sb* was then fed to mice, and the diversity of the mouse fecal microbiome was analyzed. The study identified significant changes to the microbiota in the mice treated with lysozyme-secreting *Sb* compared to the control or wild-type *Sb* group. These studies point to a special concern with using engineered probiotics for non-targeted antimicrobial biosynthesis. Although the production of these compounds is localized to the gut, the compound may have off-target effects on other commensal microbes, thus furthering gut dysbiosis and selecting for the development of resistance.

### 5.3. Production of targeted antimicrobials

The final category of molecules produced in engineered *Sb* are biotherapeutics that target pathogenic microorganisms. Li *et al*. used an engineered *Sb* to produce Leucocin C, a bacteriocin that targets *Listeria monocytogenes* [81]. Researchers showed that their Leucocin C plasmid construct was stable in the engineered *Sb* strain and enabled it to inhibit *Listeria* growth on plates and in liquid co-cultures [81]. This work may be transferable to other natural bacteriocins targeting different pathogens.

Medium-Chain Fatty Acids (MCFAs) are fatty acids that range from 8 to 12 carbons. Fatty acids of these sizes have been shown to have some activity in inhibiting the growth of *Candida albicans* [78]. Three genes necessary for MCFA biosynthesis were placed under the control of galactose-inducible or constitutive promoters and expressed in wildtype *Sb*. This pathway enabled the production of various sizes of MCFAs in the supernatant of each engineered *Sb* culture [78]. In the presence of MCFAs from both the inducible and constitutive strains, researchers observed that *Candida* exhibited reduced biofilm formation and no hyphae formation [78].

A final example of engineering *Sb* to target pathogens is the treatment of *Clostridiodes difficile* infections. *C. difficile* is an opportunistic pathogen that can cause severe disruptions to the gastrointestinal tract and has a high likelihood of recurrence after initial treatment. Additionally, this pathogen is challenging to treat due to its ability to form dormant spores and its highly antibiotic-resistant nature. In a 2020 study by Chen *et al*. [71], researchers created a tetra-specific, quadrivalent antibody (ABAB) that neutralized both toxin A (TcdA) and toxin B (TcdB) from *C. difficile*. *Sb* was then transformed with a plasmid containing the ABAB antibody expression cassette. *In vitro* studies confirmed that *Sb* secreted the secreted antibody via the α-mating factor and invertase secretion signals. Researchers then found that mice treated with the engineered *Sb* strain after a *C. difficile* spore challenge had significantly lower mortality rates, faster recovery, less tissue damage, and less inflammation in the cecum. These promising results suggest that engineered *Sb* may be used to supplement current antibiotic and antibody therapies for *C. difficile* infections.

As the groundwork for fully understanding the capabilities of *Sb* to serve as a biotherapeutic chassis is ongoing, this exciting and promising research exemplifies *Sb*’s suitability for the job. Many studies described above successfully engineered *Sb* to produce the desired product, showing promising effects in *in vitro* experiments and *in vivo* models. So far, engineered *Sb* has been used in a wide range of applications, from further enhancing its natural probiotic effects and delivering vitamins to the gut, to targeting antibiotic-resistant diseases by delivering pathogen-specific therapeutics to the site of infection. Some interesting future studies may include targeting other vitamin deficiencies [82]. While the above studies have been foundational in creating an *Sb-specific* knowledge base, a more fundamental exploration of *Sb* is needed. With these advancements, there can be continuous refinement in how the *Sb* chassis produces and delivers relevant therapeutics to help human patients.

## 6. Conclusion and future perspectives

The recent explosion of interest in using *Sb* as a drug delivery vehicle reflects its unique properties as a probiotic. As a eukaryote, it can be easily engineered to secrete high protein titers and is intrinsically resistant to antibiotics that will likely be used in conjunction with some *Sb* therapies. As a yeast, it is easy to manufacture, freeze dry, and transport long distances at room temperature, and has a long shelf life, making it highly suitable for use in resource-limited settings. It is also unable to be infected by phage and does not mate, making it less likely to participate in horizontal gene transfer. As described in this review, it is also highly amenable to genetic engineering in much the same way as its close cousin, *Sc*. Finally, although all studies to date have engineered *Sb* for use in the human gut, its engineering applications may not be limited to that environment. *Sb* has long been known to improve animal growth and reduce bacterial infections, indicating that genetic engineering could be used to further improve these properties and enhance sustainability. Recent studies have also indicated that *Sb* may be a promising treatment for vulvovaginal candidiasis and bacterial vaginosis, suggesting that additional expression of antibacterial compounds or *Candida*-binding proteins may be synergistic [83,84]. And while wild-type *Sb* had no significant impact on wound healing in a porcine model [85,86], the lack of adverse events reported in these studies indicates that engineered *Sb* may be a promising chassis for delivery of factors that accelerate wound healing and prevent infection.

However, there are some important limitations inherent to this species. First, unlike many gut bacteria, it cannot reside in the gut for long periods, at least not without further engineering. Second, it is susceptible to the same antifungals used to treat fungal infections of the gut, making it suboptimal for use in tandem with antifungals. Also, like other bacterial probiotics, it should not be used by individuals with compromised immune systems, as dangerous bloodstream infections have been observed in several cases [87,88]. By weighing the benefits and limitations of this chassis against other available probiotics, the right species (if any) for each therapeutic area may be chosen.

As of this writing, no genetically modified probiotics (including *Sb*) have been FDA-approved to diagnose, prevent, or cure any disease. Although the primary reason for this is likely the newness of the *Sb* field, additional clues may be found in the relative numbers of studies focusing on specific disease areas (many) versus general enabling technologies (few). At this stage, it seems unlikely that true therapeutic impact will be attained by repurposing the same strategies optimized in *Sc* for high production in bioreactors. Instead, a deep and thoughtful adaptation of *Sb*’s therapeutic activities to gut conditions will likely have greater success. For this reason, we are encouraged by studies that, for example, test promoter activities in gut (or gut-like) conditions. These studies could lead to the development of gene expression regulators that enable consistent dosage of engineered therapeutics despite environmental fluctuations within the intestine. We are also inspired by studies that engineer *Sb* to utilize carbon sources uniquely available within the intestine and by fundamental studies investigating the biology and lifestyle of gut-resident fungi [89], as exploitation of their lifestyle strategies (e.g. mucoadherence and metabolic collaboration with gut bacteria) may allow for the development of engineered strains with defined residence times. Combining such colonization-enhancing pathways with disease-responsive expression regulators could enable the development of *Sb* strains that proliferate only in the context of active disease, and then exit the gut once treatment is complete. We are also hopeful that the ability to perform *in situ* biomanufacturing using a variety of microbes has inspired the development of drugs that are designed to overcome the unique challenges present in the gut, for example via protease resistance, mucus penetration, and traversal of the epithelial lining. Looking forward, a gut-specific view of these core “unit operations” of drug delivery (genetic engineering, gene expression, colonization, sensing, and production) will lead to scalable, low-cost vehicles for treating many human and animal diseases.

## Figures and Tables

**Table 1:** Publications demonstrating successful secretion and surface display of recombinant biomolecules in *S. boulardii*. The signal sequence responsible for the highest titer is indicated in bold for recombinant biomolecules tested using multiple secretion signals.

Year	Delivery Type	Secretion Signal	Recombinant Biomolecule	Application	Titer	Publication
2013	Secretion	α-Mating Factor	Viral IL-10 homologs	Downregulation of inflammatory cascades	0.1–0.9 mg/L (*in vitro*), 1.4–1.9 mg/L (colitis mouse model)	[69]
2014	Surface Display	Aga1	eGFP, EtMIC2	Both molecules used for surface display detection via fluorescence or immunofluorescence	Not Reported	[43]
2016	Secretion	Chicken lysozyme	Human lysozyme	Hydrolyzes 1,4 glycosidic linkages in N-acetylmuramic acid and N-acetylglucosamine	~450 units/mL	[29]
2018	Secretion	α-Mating Factor	Ovalbumin fusion protein	Stimulation of IgG and IgA in murine model	4.7 mg/L	[70]
2020	Secretion	α-Mating Factor, Invertase	ABAB antibody	Tetra-specific antibody against *C. difficile* TcdA and TcdB	Not Reported	[71]
2020	Secretion	α-Mating Factor	IL-10, TNFR1-ECD, Alkaline phosphatase (AP), Atrial Natriuretic Peptide (ANP)	Reducing inflammatory immune response in the gut	4E-5 mg/L (IL-10)	[72]
2021	Secretion	α-Mating Factor	Leucocin C	Bacteriocin inhibiting *Listeria monocytogenes*	Not Reported	[73]
2021	Secretion	α-Mating Factor, chicken lysozyme, Sta1, **Sed1**	Neoagarooligosaccharides	Anti-obesity, anti-diabetic, anti-inflammatory, anti-viral, and antitumor activities	1860 mg/L	[74]
2021	Secretion	α-Mating Factor	Apyrase	eATP detection and dephosphorylation, linked to inflammation response in the gut	Not Reported	[65]
2022	Secretion	Not Reported	TAN-1612	Nonantibiotic fungal anhydrotetracycline	6.1 mg/L	[75]
2023	Secretion	α-Mating Factor (full), α-Mating Factor (pre), Suc1, Yap3-TA57, **preOST1pro α-MF**	Toxin A neutralizing peptide	10-mer peptide targeted against *C. difficile* TcdA	2297 mg/L	[42]
2023	Secretion	α-Mating Factor	Exendin-4	Glucagon-like peptide-1 receptor agonist	15 nM/OD600	[76]
2023	Secretion	Chicken lysozyme	Human lysozyme	Hydrolyzes 1,4 glycosidic linkages in N-acetylmuramic acid and N-acetylglucosamine	1.1 mg/g cecal contents, 0.2 mg/g colon contents	[77]
2023	Secretion	Not Reported	Medium-chain fatty acids	Reduction of biofilm and hyphal formations in *C. albicans* SC5314	10.5 mg/L	[78]
2023	Secretion	α-Mating Factor	Polypeptide-p	Responsible for decreasing blood sugar levels by activating glucose metabolism and inhibiting intestinal absorption of glucose	Not Reported	[79]
2024	Surface Display	Aga2	Aga2-monomeric streptavidin	Provides location for surface binding of biotinylated anti-ECM antibodies	Not Reported	[44]
2024	Secretion	Not Reported	L-Ergothioneine	Antioxidant protecting against stress-induced sleep and neuronal injury caused by amyloid β	17.5 mg/L	[33]

## Data Availability

No data was used for the research described in the article.
